# Stressing Out from the Flu: A Case of Influenza A-associated Transient Cardiomyopathy

**DOI:** 10.7759/cureus.4918

**Published:** 2019-06-17

**Authors:** Erika L Faircloth, Sarfaraz Memon

**Affiliations:** 1 Internal Medicine, University of Connecticut, Farmington, USA; 2 Cardiology, Hartford Hospital, Hartford, USA

**Keywords:** influenza, stress-induced cardiomyopathy, takotsubo cardiomyopathy, cardiology

## Abstract

Influenza infections are prevalent and have a large impact on our health system. They are associated with multiorgan complications that can have significant morbidity and mortality. Although influenza is a known etiology of myopericarditis, only a few case reports have documented influenza as a cause of takotsubo cardiomyopathy or stress-induced cardiomyopathy. We present a patient who developed a new left bundle branch block with positive cardiac markers, nonobstructive coronary arteries and a new cardiomyopathy that reversed within 48 hours of diagnosis of influenza A infection. This case highlights a rarer cardiovascular complication of influenza; one that would dictate medication changes, require close follow-up, and have a possibility of recurring.

## Introduction

Influenza is a single-stranded virus of the Orthomyxoviridae family classified by antigenic differences in proteins [1]. Influenza infections are prevalent and have a large impact on our health system. It is estimated that there were 49 million cases of infections, 960,000 hospitalizations, and 79,000 deaths due to influenza in the United States from 2017-2018. There are multi-organ complications associated with the infection including superimposed pneumonia, encephalitis, myositis, myocarditis, sepsis and death [2].

Takotsubo cardiomyopathy (TCM), or stress-induced cardiomyopathy, is a condition in which there is ephemeral left ventricular hypokinesis, often apical, in the setting of either physical or emotional stress [3,4]. Although myopericarditis is a known complication of influenza, only a few case reports have documented TCM due to influenza infection [5]. We present a case of stress-induced cardiomyopathy secondary to the influenza A infection. Our case highlights the importance of considering cardiovascular complications of influenza as management of heart failure and close follow-up are warranted.

## Case presentation

A 59-year-old woman presented to an outside hospital with dysuria, weakness, and diarrhea for 10 days prior to presentation. Her pertinent medical history included hypertension, hyperlipidemia, coronary artery disease status post drug eluding stent to the right coronary artery with residual 60% disease in the left anterior descending artery, chronic obstructive pulmonary disease, and craniopharyngioma status post resection complicated by pan-hypopituitarism. She was an active smoker with a 40 pack-year history but denied alcohol or illicit substances.

Initial examination included a normal blood pressure, pulse of 46 beats per minute, temperature of 91.6 degrees Fahrenheit, and saturating 92% on room air. She had occasional scattered rhonchi on lung exam and no murmurs, rubs or gallops on cardiac exam. She did not have jugular venous distension, and no peripheral edema. Chest X-ray was without evidence of acute disease and electrocardiogram (ECG) revealed sinus bradycardia. The patient had leukopenia to 2.1 thou/uL and thrombocytopenia to 42 thou/uL. Electrolytes, lipase and lactic acid were normal. Urinalysis was supportive of a urinary tract infection and she was started on antibiotics.

**Figure 1:**
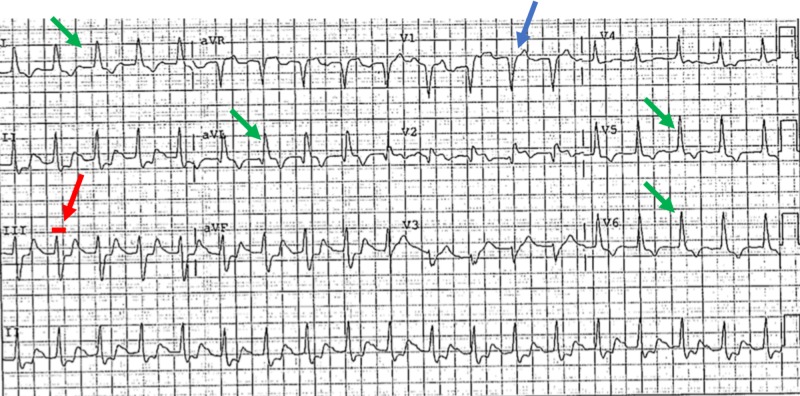
ECG revealing a new LBBB evidenced by: QRS duration of >120 ms (red arrow), lead V1 with a dominant deep S wave (blue arrow), broad, monophasic R wave in the lateral leads with absence of Q waves (except aVL) (green arrows). ECG: Electrocardiogram; LBBB: Left bundle branch block; ms: milliseconds.

On day three of hospitalization, the patient developed sudden onset of shortness of breath requiring bi-level positive airway pressure ventilation. A computed tomography angiography of the lungs was performed and ruled out pulmonary embolism. Troponins were drawn and were elevated. A repeat ECG was performed (Figure 1) revealing a new left bundle branch block (LBBB).

Four hours later, the LBBB resolved. An echocardiogram was performed which demonstrated a new reduction in ejection fraction (EF) of 20% with the wall motion abnormality most prominently in the territory of the left anterior descending artery. There was no apical ballooning noted. At this point, the patient was transferred to our hospital for further management of the new cardiomyopathy with positive cardiac markers.

On arrival, influenza testing was performed and the patient was positive for influenza A infection for which she was started on oseltamivir. The patient then underwent angiography which revealed non-obstructive coronary artery disease. A repeat echocardiogram was then performed within 48 hours of prior with a demonstrated EF of 61%.​ The patient’s symptoms improved and she was discharged home with close follow-up with her primary care provider.

## Discussion

In the early 1930s, clinicians started to note an association between patients presenting to their offices with upper respiratory illnesses and subsequent cardiovascular events [6]. Since that time, studies have shown higher prevalence of cardiovascular mortality during times of influenza epidemics [1]. In a self-controlled case-series by Kwong et al., there was a statistically significant association between documented influenza infections and acute myocardial infarction admissions within the first seven days after diagnosis. Infectious illnesses may provoke acute coronary syndrome (ACS) in those with underlying atherosclerotic disease by way of inflammation, vasoconstriction, platelet activation and endothelial dysfunction. ACS can also be triggered by an increase in metabolic demand leading to hypoxemia [6].

TCM or stress-induced cardiomyopathy was first discovered in Japan in the 1990s and is predominantly seen in elderly women [3,5]. TCM can present like ACS, however, on examination of the coronaries, obstructive disease cannot be demonstrated [7]. In patients who are suspected to have ACS, studies have shown that 1.0-2.2% of cases are related to stress-induced cardiomyopathy [1,8]. A review of 14 studies on takotsubo cardiomyopathy by Gianni et al. revealed that 81.6% of patients had ST elevation, usually in the precordial leads, 64.3% of patients had T wave abnormalities and 31.8% of patients had Q wave abnormalities. In the same review, 86.2% of patients had elevated troponin, and 73.9% had elevations in CK-MB [8]. The typical findings on echocardiography are apical ballooning with hypokinesis seen in the basal segments which can resemble an octopus pot, however, there are variations to this [4]. Abnormal ventricular function tends to resolve within days to weeks [3,8]. The exact mechanism of TCM is not fully elucidated, however, the most accepted hypothesis is that there is dysautonomia secondary to catecholamine discharge [4]. This is supported by prior studies finding a significant increase in measured catecholamines in patients hospitalized with TCM comparative to those with ACS [8].

Influenza is known to cause myopericarditis in about 10% of cases, but only a few case reports have reported stress-induced cardiomyopathy triggered by the virus [3-5,9]. Cioni reported an unfortunate case of TCM secondary to influenza which was complicated by cardiac arrest highlighting the potential dangers [7]. Buzon et al. reported new stress-induced cardiomyopathy due to the H1N1 subtype of influenza [5]. Myocardium infected with influenza A have an increased expression of TNF-a, TNFRI and TNFRII. Through nitric oxide and calcium handling pathways, TNF-a can depress the contractility of myocardial tissue [1]. Although TCM is almost always reversible in weeks, there is up to a 2.9% risk of recurrence annually for the first four years following the initial event [5]. While the patient’s ventricular function is improving, beta blockers, angiotensin converting enzyme inhibitors and diuretics can be used [3].

## Conclusions

Influenza infections can have severe complications leading to morbidity and mortality. Cardiovascular complications include ACS and myopericarditis. We present a rarer complication, stress-induced cardiomyopathy or TCM. Although our patient recovered her ventricular function prior to discharge, some patients take weeks to recover which may require medical management of the reduced ejection fraction and close follow-up. In addition, those that develop TCM have a risk of recurrence in the future. Practitioners should be cognizant of the possibilities of influenza complications and screen for them if symptoms warrant.
